# Gender Differences in White Matter Microstructure

**DOI:** 10.1371/journal.pone.0038272

**Published:** 2012-06-06

**Authors:** Richard A. Kanaan, Matthew Allin, Marco Picchioni, Gareth J. Barker, Eileen Daly, Sukhwinder S. Shergill, James Woolley, Philip K. McGuire

**Affiliations:** 1 Department of Psychological Medicine, King’s College London, Institute of Psychiatry, London, United Kingdom; 2 Department of Psychosis Studies, King’s College London, Institute of Psychiatry, London, United Kingdom; 3 St Andrew’s Academic Centre, King’s College London, Northampton, United Kingdom; 4 King’s College London, Institute of Psychiatry, Centre for Neuroimaging Sciences, London, United Kingdom; 5 Department of Developmental Psychiatry, King’s College London, Institute of Psychiatry, London, United Kingdom; West China Hospital of Sichuan University, China

## Abstract

**Background:**

Sexual dimorphism in human brain structure is well recognised, but little is known about gender differences in white matter microstructure. We used diffusion tensor imaging to explore differences in fractional anisotropy (FA), an index of microstructural integrity.

**Methods:**

A whole brain analysis of 135 matched subjects (90 men and 45 women) using a 1.5 T scanner. A region of interest (ROI) analysis was used to confirm those results where proximity to CSF raised the possibility of partial-volume artefact.

**Results:**

Men had higher fractional anisotropy (FA) in cerebellar white matter and in the left superior longitudinal fasciculus; women had higher FA in the corpus callosum, confirmed by ROI.

**Discussion:**

The size of the differences was substantial - of the same order as that attributed to some pathology – suggesting gender may be a potentially significant confound in unbalanced clinical studies. There are several previous reports of difference in the corpus callosum, though they disagree on the direction of difference; our findings in the cerebellum and the superior longitudinal fasciculus have not previously been noted. The higher FA in women may reflect greater efficiency of a smaller corpus callosum. The relatively increased superior longitudinal fasciculus and cerebellar FA in men may reflect their increased language lateralisation and enhanced motor development, respectively.

## Introduction

Sexual dimorphism in human brain structure is well described, recently by studies using magnetic resonance imaging (MRI), with differences garnering enormous interest as potential substrates or consequences of the cognitive and behavioural differences between the genders[1]–[3]. Male brains are larger both overall and in most regions, though regional differences vary in their direction and may disappear entirely when corrected for whole brain volume[3]–[5]. When considered by tissue type, both grey and white matter raw volumes are larger in males[6]–[8]; but after correction for brain size, the situation is more complicated, with most studies finding relative grey matter volume to be higher in women[7]–[9], though not all studies agree [6], while relative white matter remains higher in men [7], [8], [10], [11]. As a result, the grey to white ratio is higher in women [12], and it is the white matter volume which depends more on gender than simply brain size [7].

Given the particular dependence of white matter on gender, the question follows as to how this difference is sustained at the microstructural level: how relatively small white matter in women subserves the connectivity of its relatively large associated grey matter. While *post mortem* studies have revealed gender differences in grey matter neuronal density, the direction of this difference varies with the region studied [13], [14], and such studies are inevitably limited by small numbers and a focussed region of examination.

One way to examine large numbers of subjects, over all of white matter, is to use an *in vivo* imaging modality that is sensitive to white matter microstructure, such as diffusion-tensor magnetic resonance imaging (DTI) [15]. DTI uses an MRI sequence sensitised to the diffusion of water and by acquiring a measure of the diffusion in all directions inferences about the microstructure of white matter can be drawn [16]. Though it is not a direct measure of any single microstructural feature, the extent to which the diffusion follows the principal diffusion direction (the fractional anisotropy (FA)), for example, is informative about the cellular architecture and myelination of the white matter in that area [17]. This has been of great service to clinical research, being sensitive to changes too subtle to be detected with volumetric MRI, wrought either directly by white matter pathology, such as diffuse axonal injury [18], or induced by pathology in the grey matter it subserves [19]. It has also found application in the quantification of differences in normal human white matter, for example in charting the healthy development of white matter in adolescence [20] or the strengthened cortical connections that result from training [21].

DTI thus offers great promise as a medium for the investigation of white matter differences that may be small, such as those of sexual differences in the regional difference and lateralisation of grey and white matter [1]. A number of studies[22]–[36] have used DTI to compare white matter microstructure between genders but these have tended to focus on particular regions of interest (ROIs), or have been limited by small samples. As the differences appear subtle, and to vary with region, we sought to extend the previous studies, and assess the extent and size of any gender differences in FA across the whole brain on a large, well-matched sample using an optimised sequence. Such whole brain methods are particularly prone to artefacts that arise when structures that are adjacent to the ventricles are assessed, in what is known as partial-volume effect [37]. We therefore supplemented the whole brain analysis with a confirmatory ROI analysis in areas where cerebrospinal fluid (CSF) contamination was thought to be a significant possibility.

## Methods

### Subjects

One hundred and thirty-five healthy control subjects, 90 male and 45 female, were selected from a larger register of subjects recruited from the local population in south London. The gender groups were matched for age, handedness (converted to a dichotomous right/left rating from the Annett [38] or Edinburgh [39] scales, or by self-report), years of education, and intelligence quotient ((IQ, normalised from either the WASI [40], the WAIS-III [41], or the NART [42]) see table 1). The study was approved by the Bethlem and Maudsley research ethics committee and all subjects gave written informed consent.

**Table 1:** Subject demographics.

	Men (n = 90)	Women (n = 45)	p-value	test
Mean Age (SD)	25.0 (6.1)	24.1 (7.9)	0.12	Mann-Whitney
Handedness (R/L)	86/4	44/0	0.30	Fisher’s Exact
Years Education	14.8 (2.9)	16.1 (1.7)	0.29	Mann-Whitney
Mean IQ (SD)	109 (12)	110 (12)	0.61	t-test

SD: standard deviation.

### Image Acquisition

All scans were acquired on the Institute of Psychiatry’s GE Signa LX 1.5 Tesla scanner. A standard quadrature birdcage head coil was used for both RF transmission and magnetic resonance signal reception. Localiser scans were axially aligned to the AC-PC line. Images with and without diffusion weighting were acquired with a multi-slice EPI sequence over 60 contiguous 2.5 mm-thick axial slice locations. Data were acquired with a 96×96 matrix over a 24 cm field of view, yielding isotropic (2.5×2.5×2.5 mm) voxels, and data were zero-filled to 128×128, giving an apparent in-plane voxel size of 1.875×1.875 mm. The acquisition was cardiac-gated using a pulse oximeter attached to the subject’s finger. The TR was 15 cardiac R-R intervals with a TE of 107 ms. The sequence was optimised for measurement of brain parenchyma, with seven b0 images and 64 diffusion-weighted images with gradient directions uniformly distributed in space acquired at each slice location, and a maximum diffusion weighting of 1300 s mm^−2^ [43]. Acquisition time was 20–30 minutes, dependent on the subject’s heart rate.

### Image Processing

The seven b0 images for each subject were first averaged to create a reference image, then each of that subject’s diffusion-weighted images registered to their reference image based on their Mutual Information [44]. Images were masked using the Brain Extraction Tool (FMRIB, Oxford, UK). The diffusion tensor was then calculated at each voxel using multivariate linear regression after logarithmic transformation of the signal intensities [15], and FA calculated at each voxel [45] using in-house software. The images were visually inspected and excluded if there were gross anatomical abnormalities or image artefacts that would complicate further processing.

Normalisation involved a two-stage process, creating a study-specific template, and then registering FA images to it. The mean b0 image from every subject was first registered using SPM2 (Wellcome Functional Imaging Laboratory, London, UK) to the SPM2 EPI template. The derived mapping parameters for each subject were then applied to that subject’s FA image. These normalised FA images were averaged and then smoothed with an 8 mm Gaussian kernel to create the study-specific template. The original FA images were then registered to this new template using the non-linear registration in SPM2. The registered FA images were also segmented and these probabilistic maps thresholded at 10% probability to generate a liberal white/rest-of-brain mask. The FA images were smoothed with a 5 mm (full-width half-maximum) kernel, before masking with the white-matter mask, to create white-matter-only FA maps. The smoothing was simply to increase signal-to-noise ratio and minimise the effects of any residual misalignment after normalisation, though it also served to sensitise the analysis to structures with spatial extents of this size [46]. Note that, unlike the VBM analyses from which much of our VBA methodology is derived, no equivalent to the ‘modulation’ step is necessary for DTI data, as (unlike the tissue density/volume of VBM) the quantitative metrics being analysed do not have any underlying relationship to size, and are therefore not affected by the scaling which is part of the registration process.

### Voxel-Based Analysis

The principal analysis was a voxel-based ANOVA of white matter FA, conducted using XBAM v4 (Institute of Psychiatry, London, UK). The one-way ANOVA was fitted to each voxel of the normalised, segmented FA maps, using gender as the grouping variable. The ANOVA was only fitted at voxels where all subjects contributed; when combined with the thresholding described above, this confined analysis to the body of the white matter. After fitting the ANOVA model to the observed data, the subject labels were randomly permuted between the two groups to achieve the null hypothesis of no main effect of group membership on fractional anisotropy. This permutation was carried out 1000 times at each voxel - 1000 being sufficient to generate a conservative voxel-level null distribution of fractional anisotropy differences [47]. After determination of those voxels showing significant effects at a relatively low (p<0.01) threshold, sets of spatially contiguous supra-threshold voxels were identified, and the sum of the supra-threshold voxel-wise test statistics, or “mass”, of each three-dimensional cluster was calculated. The mass of each cluster was then tested against the corresponding permutation distribution. Cluster-wise probability-thresholds were chosen to ensure less than one false-positive in the imaging volume. The identification of clusters with white matter tracts was made by reference to Mori et al (2005) & Crosby et al (1962). When clusters lay adjacent to a tissue boundary with CSF (so that partial volume artefact was felt to be a significant possibility) a subsequent ROI analysis was performed to corroborate the findings.

### ROI Analysis

ROIs were defined by an independent rater, blind to gender, using a pre-defined octagonal shape of fixed size placed on each of 4 sequential slices of each subject’s native-space FA maps, using criteria previously developed for the purpose [48]. After training, the intra-rater reliability (assessed by intraclass correlation of repeated analysis of 10 scans) was 0.99. For each subject’s ROI, FA values were extracted at each voxel, and the means of these compared at the group level using SPSS 16.0 (SPSS Inc., Chicago, IL).

## Results

**Figure 1:**
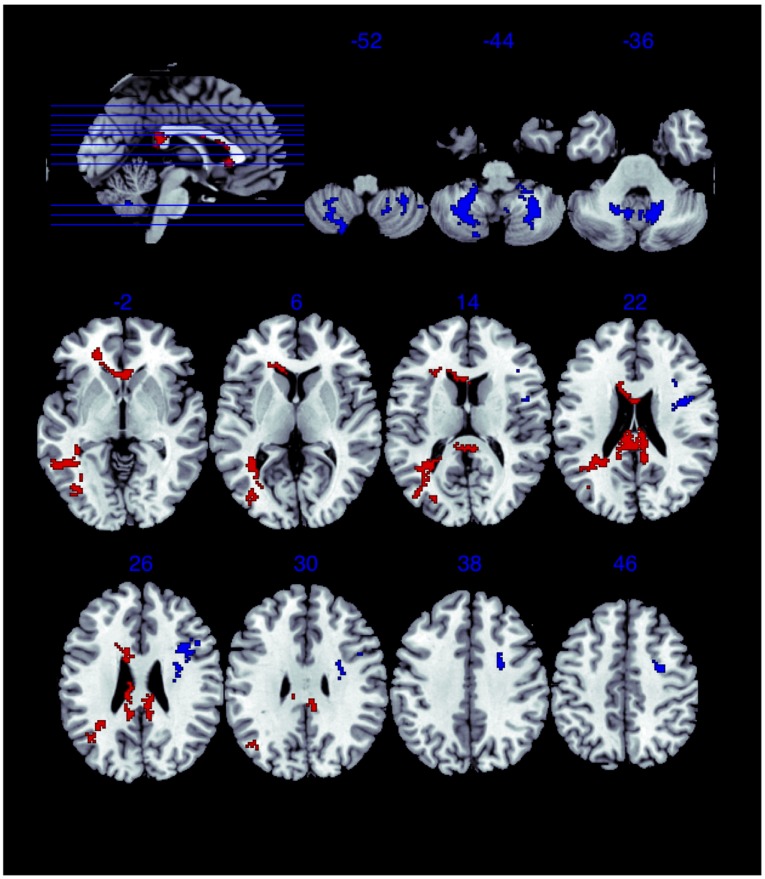
Areas of Gender Difference in Fractional Anisotropy. Red areas represent higher FA in women; Blue areas represent higher FA in men. Numbers Refer to the MNI Z-coordinate of the image below. The Left side of the brain is depicted on the Right side of the images.

The primary analysis found men to have higher FA in the cerebellum, and an area at the anterior portion of the left superior longitudinal fasciculus; women to have higher FA in the corpus callosum (see table 2, figure 1). Repeating the analysis with age or handedness as a covariate did not change the significance of the clusters. Since the corpus callosum cluster was peri-ventricular, an ROI analysis was performed to exclude partial volume effect: this confirmed the higher corpus callosum FA in women (women: mean 0.762, standard deviation 0.055; men: mean 0.744, standard deviation 0.048; Mann Whitney U test, after Kolmogorov-Smirnov test for normality: p = 0.016).

**Table 2:** Areas of FA difference.

Direction	Size	X	Y	Z	P-value	Tract
Women>Men	439	0	22	−2	0.00038	Genu of Corpus Callosum
	1241	4	−28	20	0.00003	Body of Corpus Callosum
Men>Women	1165	−25	−48	−44	0.00007	Left Cerebellum
	352	−32	13	24	0.00029	Left Superior Longitudinal Fasciculus

X, Y, Z-coordinates refer to the centre of mass of the cluster. SLF: superior longitudinal fasciculus.

## Discussion

Our whole-brain analysis revealed significant sexual dimorphism in the white matter of healthy humans’ brains, with areas showing increased FA in men compared with women and vice versa. While FA may have a number of determinants, even in healthy subjects, the principle factors are myelination and tissue architecture [17], with the inference that higher FA represents more “efficient” white matter organisation. This inference is supported by correlations of FA with conduction speed [49], [50]. These gender differences may thus be informative about function. They are also important because they are comparable with those found in some pathologies - the difference reported here is larger than some schizophrenia studies report using the same methodology, for example [48], [51], [52]. As gender is often an unbalanced factor in pathological samples it remains a potentially significant confound.

An important consideration in the interpretation of these findings is associated volume differences. Though we did not consider volume, volumetric differences have been previously described in multiple studies - even if they do not agree on the detail [1], [3]. There are two aspects to this: on the one hand, a difference in gross anatomical volume in the tract or subtended grey matter may be considered a confounder; on the other, it may be considered a potential explanatory factor. One theoretical source of confounding arises from partial volume effect, where larger volumes increase FA as the influence of partial volume diminishes [53]. This is particularly a problem for tractography methods [37], [54], however, and while it cannot be excluded in our voxel-based approach, it should be minimised by the ROI confirmation. Another concern may be over the meaning of the FA measure where volume differs, but as FA is an absolute value it would not make sense to ‘dilute’ it to represent differences in volume as is done with proton density, for example. Alternatively, the correlations of FA with white matter volume which have been found[53], [55]–[57] may reflect an overlap in the biophysical determinants of those measures, such as myelination or fiber density [55]. Equally, variations in the volume of subtended grey matter may provide an explanation in terms of the amount of connectivity ‘work’ that the white matter connections need to do. However, as the correlations between volume and FA are subtle and vary in direction across locations [55], and as the nature of any regional volume differences are themselves subtle and without agreement across studies [6], [8], [58], it is not possible to give a single prescription for the nature of the relationship and each cluster shall be considered in its own context in what follows.

We should first recognize that the location of the clusters, represented by the cluster centre in Table 2, is more complex than that table would suggest. As shown in figure 1, they are extensive: in women, they potentially extend into the anterior corona radiata, cingulum, right inferior longitudinal fasciculus, superior longitudinal fasciculus, and fronto-occipital fasciculus; in men, the left anterior cluster extends into the shorter association fibres and superior corona radiata, the cerebellar cluster includes parts of the middle cerebellar peduncle, but also intra-cerebellar white matter. Some of these findings have precedents in the small literature on gender in DTI, but some are new.

The corpus callosum has been the focus of much gender-based brain research, with studies confirming larger volumes in men [59], reflecting associations with gender differences in lateralisation [26]. Previous attempts to find an associated microstructural difference have yielded varying results, however, with some studies finding higher anisotropy in men [24], [26], [30], [34], some finding no difference [28], [33], [35], [60], one finding a division between truncus increases in men, and genu/splenium increases in women [22], and one finding increased FA in adolescent females [61]. Our study found an increase in FA throughout the corpus callosum in women, confirmed in the genu by ROI. Reconciling these findings is not easy, however only one of the previous studies was of comparable size to the present study, the other three being considerably smaller. Additionally, the other studies largely used briefer, non-optimised sequences[24]–[29], [34], where signal-to-noise ratio would be lower, further decreasing power for an already small effect size [43]. Demographic differences between the various study samples may yield other sources of variation [34], of course, but when interactions with age [28], [29] or handedness [24] have been sought, they have not been shown to affect the corpus callosum. The relative power of our study, and the combination of two analytic techniques, gives us some confidence in the validity of our findings; and the increased efficiency implied by increased FA finds confirmation in the strengthened callosal connectivity in women shown using graph theory [62], [63], and the reduced interhemispheric transfer time found [64], [65]. The smaller size of the female corpus callosum would not appear to represent disadvantage, given these findings, and may even be correlated with efficiency [23], [66].

Increased FA in the superior longitudinal fasciculus in men has not previously been noted, to our knowledge. It might be expected if widely reported gender differences in the lateralisation of language were true [1], [3], [67], [68], the greater left-dominance of male language function reflected in enhanced connectivity of left language pathways. Lateralisation effects on the FA of the superior longitudinal fasciculus have been previously reported, but their association with gender has not reached significance in two smaller [69], [70], and one much larger [71] study. A recent paper by Powell and colleagues [72] presented the opposite result, however, finding a rightward asymmetry in males in a similar area – though one that did not survive stringent multiple comparisons control. This study thus represents a potentially important confirmation of a predicted finding, one that has proven difficult to establish in gross anatomical terms [1], [73]. A lateralisation explanation, however, would also predict a relative increase in the corresponding FA of the right superior longitudinal fasciculus in female subjects, which was not found in this study. While type 2 error is a possibility, even with our relatively large sample, this does challenge the lateralisation explanation. An alternative would be the simple effects of handedness. Male subjects are more commonly non-right handed [73], as was the case in our sample, and though we controlled for those classed as left-handed, a more subtle impact of those with more even-handedness cannot be excluded. The study by Powell et al was balanced for handedness, which offers some support for this interpretation, though they did not find an interaction between handedness and gender in their own sample’s asymmetry [72]. Lastly, an explanation in terms of associated volume should be considered, as left temporal increases in grey matter volume have been noted [1]. The two largest studies [6], [8] do not agree, however, which makes an association difficult to sustain.

Sexual dimorphism is much less studied in the cerebellum than the corpus callosum. Studies generally report larger cerebellar volumes in men[4], [8], [74]–[76], though some have found this to be a function of brain volume [77], [78], with one study finding women to have a larger cerebellar volume relative to their total brain volume [79]. We are aware of only two much smaller studies that have considered gender differences in cerebellar microstructure, neither reporting differences [30], [33]. Though a correlation between cerebellar volume and FA changes in childhood has not been shown [80], cerebellar white matter development continues throughout childhood [81], and gender volume differences are already detectable in children, where they are strongly correlated with motor-cognitive function [82]. Gender differences in motor function are well described [83], and it is tempting to see an association between these and the FA differences, as has been shown, for example, between cerebellar volume and motor dexterity [84]. Gender motor differences vary with the test [83], and evolve with age [85], however, so volumetric associations are not always found [86]. A developmental correlate of cerebellar white matter organisation with motor-cognitive function seems plausible, though it has yet to be demonstrated by a targeted study.

### Conclusion

We report gender differences in microstructure in a number of white matter tracts: in the corpus callosum, women showed increased FA; while men showed increased FA in the left superior longitudinal fasciculus and cerebellum. In each case, these can be presented as plausible microstructural correlates of gender differences reported in other fields – inter-hemispheric connectivity, lateralisation, and motor development – though future studies would be required to confirm any correlation directly. Our results also conflict with some other studies from this small field. While our relatively large, well-matched sample, image quality and analysis method give us confidence in our findings, larger studies yet, using methods such as TBSS [87] that are less susceptible to volumetric confounds, may be required to give a definitive answer to the location and direction of gender differences in white matter microstructure.
